# The role of *N*-methyladenosine modification in acute and chronic kidney diseases

**DOI:** 10.1186/s10020-023-00764-w

**Published:** 2023-12-08

**Authors:** Saiqi Qi, Jie Song, Linjun Chen, Huachun Weng

**Affiliations:** The College of Medical Technology, Shanghai University of Medicine & Health Sciences, 279 Zhouzhu Highway, Pudong New Area, Shanghai, 201318 People’s Republic of China

**Keywords:** m6A modification, Acute kidney injury, Chronic kidney disease

## Abstract

*N*6-methyladenosine (m6A) modification is a kind of RNA modification in which methylation occurs at the sixth N position in adenosine in RNA, which can occur in various RNAs such as mRNAs, lncRNAs and miRNAs. This is one of the most prominent and frequent posttranscriptional modifications within organisms and has been shown to function dynamically and reversibly in a variety of ways, including splicing, export, attenuation and translation initiation efficiency to regulate RNA expression. There are three main enzymes associated with m6A modification: writers, readers and erasers. Increasing evidence has shown that m6A modification is associated with the onset and development of kidney disease. In this article, we address the important physiological and pathological roles of m6A modification in kidney diseases (uremia, ischemia–reperfusion kidney injury, drug-induced kidney injury, and diabetic nephropathy) and its molecular mechanisms to provide reference for the diagnosis and clinical management of kidney diseases.

## Introduction

Kidney disease has been ranked among the top 10 causes of death worldwide (Yang et al. 2020a). According to the *Survey Report on the Epidemiological Study of Kidney Disease* published in 2019, the total incidence of chronic kidney disease (CKD) in China is 10.8%. It is estimated that there are approximately 160 million kidney disease patients, its incidence is on the rise, and the majority of patients eventually develop irreversible end-stage kidney disease (ESKD). Therefore, the prevention and treatment of kidney disease has become a public health priority; however, the detailed pathogenesis of this disease is not clear.

The most common eukaryotic RNA modification is adenosine methylation at the 6th nitrogen position, which is known as *N*6-methyladenosine (m6A). The m6A modification, which was first discovered after the discovery of the polyadenylate (Poly A) structure of messenger RNA (mRNA) in the 1970s, is considered to be the most prevalent and abundant posttranscriptional modification in eukaryotic mRNAs, microRNAs (miRNAs), long noncoding RNAs (lncRNAs) and circular RNAs (circRNAs) (Desrosiers et al. 1974; Schafer et al. 1982; Rottman et al. 1974; Yang et al. 2017; Chen et al. 2019a). m6A modifications are mainly enriched in the 3ʹ-untranslated regions (UTRs) around the mRNA stop codon and play critical roles in mRNA splicing, nuclear export, degradation and translation to control changes in gene expression (Chen et al. 2019a; Dominissini et al. 2012; Livneh et al. 2020). Numerous studies have shown that abnormal m6A methylation can lead to developmental disorders, nervous system diseases, cardiovascular diseases, kidney diseases, and tumor occurrence (Li et al. 2021a, b, c, d, e; Jiang et al. 2022a; Kumari et al. 2022; Cui et al. 2021). The relationship between m6A and kidney diseases has been intensively studied in recent years, and a variety of m6A-related biomarkers have been used in the diagnosis and treatment of kidney diseases (Wang et al. 2022; Meng et al. 2020; Sun et al. 2022; Zhou et al. 2019). In this article, we review the roles and mechanisms of m6A regulatory changes in the development and prevention of kidney diseases to provide a reference for research on the prevention and treatment of kidney diseases.

## M6A-related enzymes

There are three main enzymes related to m6A modification: writers, readers and erasers. These enzymes add, remove and recognize m6A sites. Adenosine undergoes methylation at the sixth N via enzymes called methyltransferases. Among them, Methyltransferase Like 3 (METTL3) was the first identified component of the m6A methyltransferase complex, which mainly acts as the catalytic core; Methyltransferase Like 14 (METTL14) is the other active component of the complex, which serves as the structural support for RNA binding. These two factors form the complex in a 1:1 ratio (Liu et al. 2014). Recombinant Wilms Tumor 1 Associated Protein (WTAP) is the third significant component of the complex, which although not catalytically active for m6A modification, acts as a bridging protein to interact with both factors, thereby affecting RNA loading and recruiting other m6A methyltransferases (e.g. KIAA1429) (Ping et al. 2014).

Methylated modified RNA base sites require specific enzymes to be recognized. These enzymes are known as m6A recognition proteins, and the most common are members of the YTH N6-methyladenosine RNA binding proteins (YTHDF) family including YTHDF1, YTHDF2, and YTHDF3 (Luo and Tang 2014). These enzymes can play a role in recognizing bases at which m6A methylation occurs. They can participate in downstream translation, degrade mRNA, and accelerate mRNA exit from the nucleus. Among them, YTHDF1 has been shown to be involved in the cross-priming of tumor antigens in dendritic cells and the cross-activation of CD8^+^ T cells (Han et al. 2019). In human cancers, YTHDF1 is more highly expressed than in normal tissues and plays an important role in the tumor microenvironment, participating in immune regulation (Hu et al. 2021).

M6A markers can also be cleared by RNA demethylases, including fat mass and obesity-associated protein (FTO) and ALKB homolog 5 (ALKHB5) (Chen et al. 2019a). FTO was the first demethylase identified regulates selective shearing. ALKBH5, on the other hand, removes methylation modifications mainly through physiological interactions with m6A (Zheng et al. 2013).

In recent years, an increasing number of studies have shown that m6A methylation is closely related to physiological activities and that abnormal m6A modification is the cause of many diseases, especially kidney diseases such as diabetic nephropathy (DN), podocyte disease, and renal cancer. In this review, we focus on the mechanism of m6A methylation in acute and chronic kidney diseases to provide help for further clinical diagnosis and treatment.

## Acute kidney injury (AKI)

**Fig. 1:**
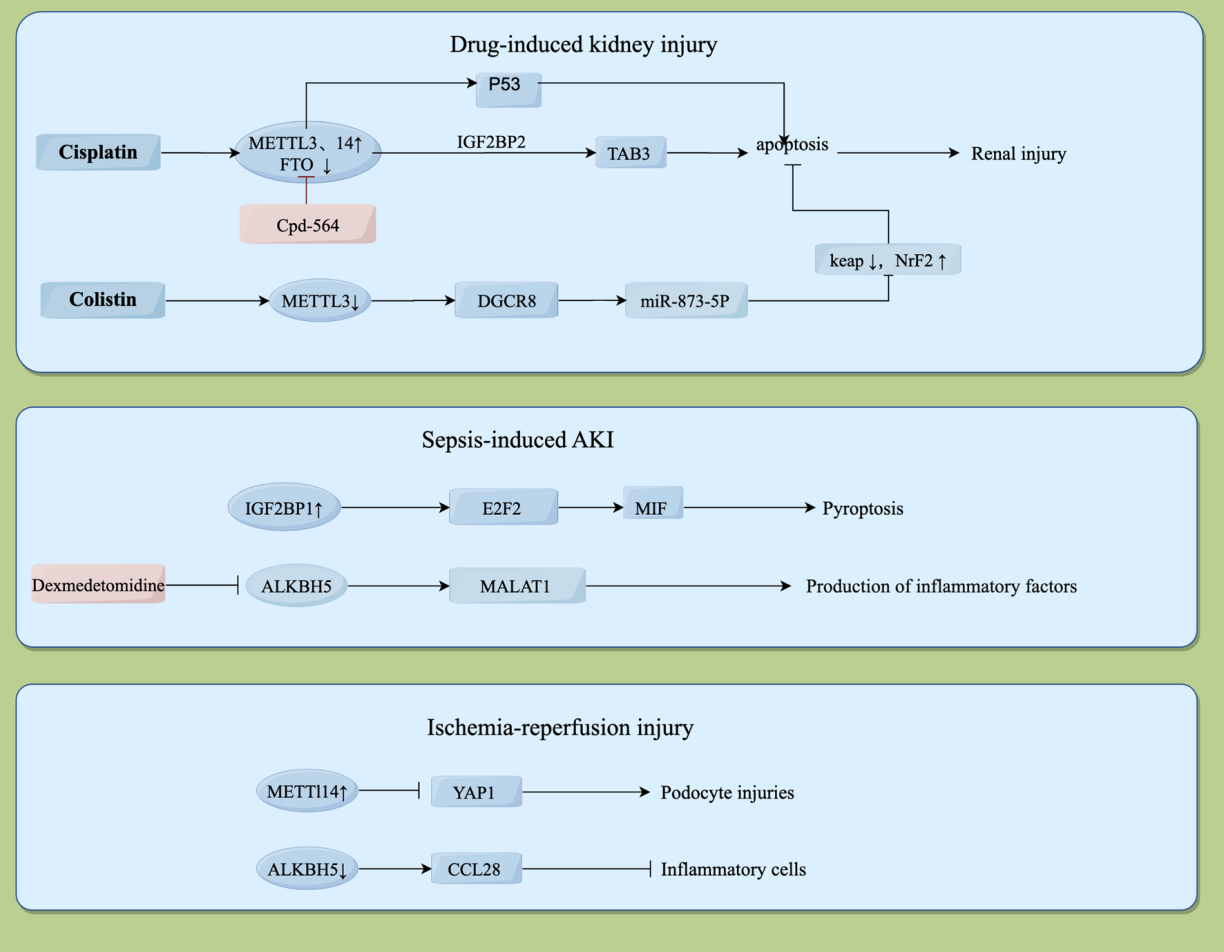
The function of m6A methylation in acute kidney injury. Pink marks indicate the intervention for methylation-related enzymes and possible use for disease treatment. ↑ indicates up-regulation; ↓ indicates down-regulation

AKI is a group of syndromes characterized by a sudden decline in renal function, accompanied by significant mortality rates and increasing hospitalization rates. AKI can trigger or develop into CKD, and eventually develops into ESKD. Exploring the molecular pathogenesis of the early AKI is critical for the development of therapeutic and diagnostic tools. Despite important advances in recent studies of AKI, the pathogenesis of AKI at the cellular and molecular levels remains incompletely unclear. Increasing evidence has shown that m6A modification is associated with AKI, as shown in Fig. 1.

## Drug-induced kidney injury

Drug-induced kidney injury accounts for approximately one-fourth of AKI cases, and nephrotoxic drugs remain an important cause of AKI in both inpatients and community patients, especially those suffering from cancer (Loghman-Adham et al. 2012; Boventre et al. 2010). The main mechanism is drug-induced renal tubular epithelial injury, which causes acute tubular necrosis.

Cisplatin is a heavy metal complex that inhibits the replication of DNA, and mainly acts on the purine and pyrimidine bases of DNA; it is widely used as a chemotherapeutic agent in the treatment of many malignancies (Hu et al. 2016). The use of cisplatin may cause AKI, which is mainly caused by changes in the expression of methylation-related enzymes such as METTL3, METTL14, WTAP, FTO, and ALKHB5, resulting in increased levels of total RNA m6A in kidney tissues after cisplatin treatment. This finding suggests that m6A plays a crucial role in the pathophysiological process of cisplatin-induced kidney injury (Li et al. 2021b). Zhou et al. found that cisplatin inhibited FTO expression, and upregulated METTL3 and METTL14, which increased m6A methylation and upregulated P53, exacerbating cisplatin-induced kidney injury through the P53-mediated Bax/Bcl-2 and Caspase3 pathways (Zhou et al. 2019). Meclofenamic acid, which is an inhibitor of FTO, exerts the same effect as cisplatin in promoting m6A methylation in HK2 cells and renal cells by inhibiting FTO expression, promoting apoptosis and thus exacerbating kidney injury (Zhou et al. 2019). METTL3 promotes the m6A modification of TAB3 through an insulin-like growth factor 2 mRNA binding protein 2 (IGF2BP2)-dependent mechanism, thereby enhancing its stability. Cpd-564, as an inhibitor of METTL3, inhibits inflammation by inhibiting the expression and abundance of TAB3. Both genetic and pharmacological inhibition of METTL3 attenuated renal injury and inflammation, suggesting that the METTL3/TAB3 axis is a potential target for the treatment of AKI (Wang et al. 2022). In addition, other researchers found that cisplatin-induced hypermethylation of Havcr1 upregulated its expression, while flavopiridol-induced hypomethylation of Hacvr1 downregulated its expression to attenuate cisplatin-induced renal injury by studying changes in the gene expression profile, providing a basis for the study of the mechanism of kidney injury and new therapies (Shen et al. 2020).

Colistin is an anti-gram-negative bacillus antibiotic with strong antibacterial effects against most gram-negative bacilli. However, the side effects of colistin have not been very clearly and accurately verified in clinical practice. Therefore, physicians have doubts about the use of colistin in clinical practice (Wertheim et al. 2013). Wang et al. found that METTL3-catalyzed m6A methylation could regulate the maturation of miR-873-5p in colistin-induced kidney injury. METTL3-dependent m6A modification could regulate the maturation process of miR-873-5p through DGCR8, a core molecule associated with miRNA precursor processing, and then regulate the Keap1/Nrf2 pathway, which in turn inhibits the proapoptotic effect of reactive oxygen species generated by oxidative stress on renal cells to protect the kidney (Wang et al. 2019).

## Sepsis-induced AKI

Sepsis is a syndrome of the systemic inflammatory response caused by the invasion of pathogenic organisms such as bacteria and approximately one-third of patients have severe AKI (Zhou et al.^2019^). Recent evidence shows that microvascular dysfunction, inflammation, and metabolic reprogramming are three fundamental mechanisms that play a role in the development of sepsis-induced AKI (Peerapornratana et al. 2019). However, the current antibiotic therapy widely used in clinical practice is not effective in treating AKI.

Zhu et al. found that dexmedetomidine reduced the production of inflammatory factors by inhibiting the expression of ALKBH5, increasing the level of m6A, and decreasing the expression of Metastasis Associated Lung Adenocarcinoma Transcript 1 (MALAT1). This study provides us with a new target for the prevention and treatment of sepsis-induced kidney injury (Zhu and Lu 2020). Insulin-like growth factor 2 mRNA binding protein 1 (IGF2BP1) is a potent pyroptosis inducer in septic AKI that targets the macrophage migration inhibitory factor (MIF) component of NOD-like receptor thermal protein domain associated protein 3 (NLRP3) inflammasomes. Inhibiting IGF2BP1 could be an alternate pyroptosis-based treatment for septic AKI (Mao et al. 2023).

Ferroptosis is a novel iron-dependent form of programmed cell death that is distinct from apoptosis, necrosis, and autophagy. The mmu-miR-7212-5p-homx1 signaling pathway was shown to promote iron death while enhancing the pathogenesis of inflammation-involved sepsis-induced AKI (Liu et al. 2022). Furthermore, METTL3 was downregulated and the reader protein ALKBH5 was upregulated in sepsis-induced AKI mice, and there were multiple m6A methylations in the Homx1 gene. The total m6A RNA methylation level might be reduced in sepsis-induced AKI and that inflammation caused by the ferroptosis pathway could be inhibited by an inhibitor of mmu-miR-7212-5p. However, the detailed mechanisms of m6A RNA methylation regulation and signaling pathways in septic AKI need to be verified by further experiments.

## Ischemia–reperfusion kidney injury

Ischemia–reperfusion injury (IRI) is an organic ischemic injury caused by insufficient blood supply to target tissue and cells due to insufficient oxygen supply under various conditions (Bonventre and Yang 2011). The methylation enzymes METTL3 and METTL14 were shown to have a close association with IRI (Meng et al. 2020; Xu et al. 2020). Foxd1 is involved in the negative regulation of cell proliferation and kidney development. It was found that Foxd1 has increased RNA m6A levels with the upregulation of METTL3 expression levels, leading to the down-regulation of its RNA, which then promotes IRI (Meng et al. 2020). Liu et al. found that METTL3 knockdown in mouse mesangial cells (MMCs) drastically reduced the levels of m6A RNA methylation. The levels of the proinflammatory cytokines IL6 and TNF-α are reduced by low m6A methylation, and inhibit cell proliferation and cycle progression (Liu et al. 2023). YAP1, a downstream target of METTL14 in IRI progression, is crucial in regulating the proliferation and differentiation of various mature cells. YAP1 is inhibited by METTL14 to promote renal AKI. Inhibition of YAP1-TEAD signaling by peptide 17 can promote renal IRI (Xu et al. 2020). CCL28 is a target of ALKBH5, and ALKBH5 deficiency increases CCL28 mRNA stability. Inhibition of ALKBH5 promotes m6A modification of CCL28 mRNA, enhancing its stability, regulating the Treg/inflammatory cell axis, and inducing Tregs, which improves renal function (Chen et al. 2023).

## CKD

**Fig. 2:**
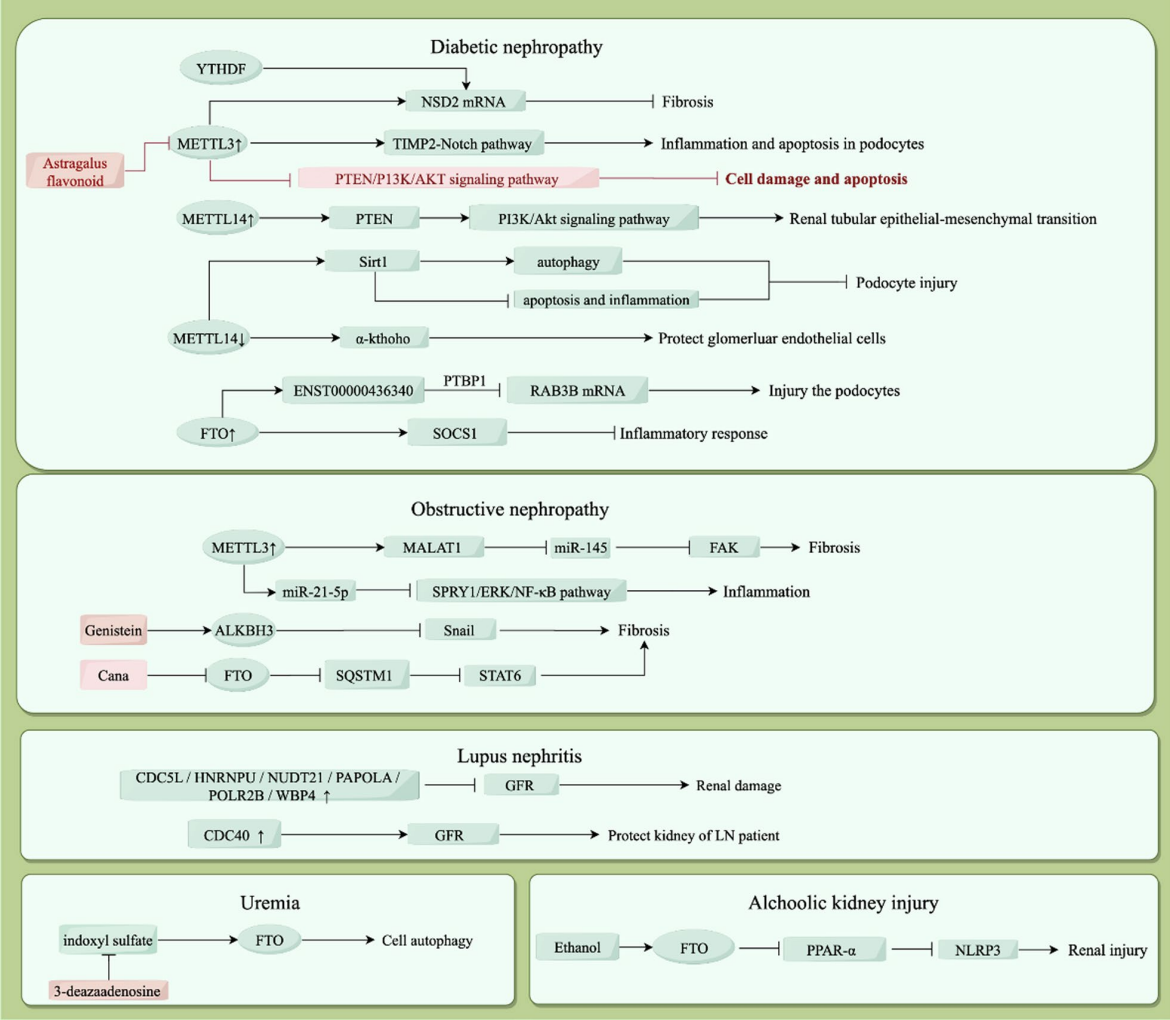
The function of m6A methylation in chronic kidney disease. Pink marks indicate the intervention for methylation-related enzymes and possible use for disease treatment. ↑ indicate up-regulation; ↓ indicate down-regulation

CKD is a major risk factor for various cardiovascular diseases. CKD is a cause of proteinuria, and proteinuria is well known to be an aggravating factor for CKD. However, the molecular mechanism of CKD remains unclear (Yang et al. 2020a). Increasing evidence has shown that m6A modification played an important role in CKD, as shown in Fig. 2.

## DN

DN is one of the most critical complications of diabetes and an important cause of CKD and renal failure (Yang et al. 2020a). Although the exact mechanism of DN is unknown, inflammation is considered to be the main phenomenon in the development and progression of the disease (Gu 2019). Current clinical treatment for DN is limited to lowering patient' blood glucose and lipids, and there are no strong or effective targeted therapeutic approaches for treating DN (Choudhury et al. 2010).

There is a close relationship between m6A modification and DN, and METTL3 has been shown to be necessary for insulin secretion (Li et al. 2021e). Moreover, m6A modification provides several promising targets for the treatment of DN, and the m6A modification-related enzymes that mainly affect the pathogenesis of DN are FTO, METTL3 and METTL14.

METTL3 has been shown to promote m6A modification of histone transferase NDS2 mRNA in mice and promote the stability of NSD2 mRNA through YTHDF1, subsequently inhibiting renal fibrosis and renal injury in DN mice (Tang et al. 2022). Additionally, METTL3-mediated m6A modification is an important mechanism of podocyte injury in DN, and can regulate inflammation and apoptosis in the podocytes through the TIMP2-mediated Notch signaling pathway (Jiang et al. 2022b). Moreover, astragalus flavonoid, which is widely used in the clinical treatment of DN, has been shown to ameliorate podocyte scorching and injury under high glucose conditions by targeting METTL3 for m6A modification thereby mediating NLRP3 inflammasome activation and PTEN/PI3K/Akt signaling (Liu et al. 2021a). These results indicate the high potential and feasibility of METTL3 as a new target for the treatment of DN.

METTL14 could play a similar role as a target in DN. Decreased METTL14-dependent RNA m6A modification could increase the stability of Sirtuin 1 (Sirt1) mRNA after transcription, thereby exacerbating podocyte injury and proteinuria. Their findings suggest METTL14-dependent RNA m6A modification contributes to podocyte injury through posttranscriptional regulation of Sirt1 mRNA, which provide a potential approach for the diagnosis and treatment of podocytopathies (Lu et al. 2021). Furthermore, when METTL14 expression is significantly reduced, the expression of α-klotho is significantly increased to protect glomerular endothelial cells from apoptosis (Li et al. 2021c). Similar to METTL3, METTL14 regulates the PI13K/Akt signaling pathway. METTL14 overexpression can increase the expression of PTEN, leading to PI3K/Akt signaling pathway inactivation in high glucose-treated HK2 cells, thus including HDAC5 downregulation and improving high glucose-induced Epithelial–mesenchymal transition (EMT) in renal tubular cells (Xu et al. 2021).

Moreover, there is increasing evidence suggesting that lncRNAs play an important role in DN (Majumdeer et al. 2019). FTO-mediated m6A modification can upregulate lncRNA ENST00000436340, enhancing the binding of PTBP1 to its target gene RAB3B by interacting with the PTBP1 protein and promoting degradation of RAB3B mRNA, which mediates cytoskeletal rearrangement and inhibits GLUT4 translocation, which leads to podocyte injury and DN progression (Hu et al. 2023). It is well known that inflammation is a key factor in DN. When FTO is highly expressed, the m6A level of SOCS1 (suppressor of cytokine signaling), a key regulator of inflammation, is significantly reduced, which increases the expression of SOCS1 and suppresses the inflammatory response thereby reducing renal injury (Sun et al. 2022).

## Lupus nephritis (LN)

LN is one of the most serious complications of systemic lupus erythematosus (SLE) and one of the most important causes of ESKD in China. The main pathogenic mechanism involves the disruption of the autoimmune system and the deposition of immune complexes formed in the glomeruli (Anders et al.^2020^).

Studies have shown that the pathogenesis of LN is closely related to m6A RNA methylation and the immune microenvironment (Zhao et al. 2021). The expression of 13 m6A regulators was downregulated in glomeruli with LN compared to healthy controls. Seven m6A methylation modification markers (CDC40, CDC5L, HNRNPU, NUDT21, PAPOLA, POLR2B, and WBP4) were associated with m6A modifications. Among them, the expression of CDC40 was positively correlated with GFR, suggesting that it may have a protective effect on the kidneys of LN patients. The other six markers were negatively correlated with GFR, suggesting that high expression of these genes may exacerbate renal damage in LN patients. Therefore, these seven factors may be potential biomarkers to determine the prognosis of LN (Zhao et al. 2021).

## Alcoholic kidney injury

Excessive alcohol consumption causes more than 3 million deaths each year, accounting for 5.3% of all deaths worldwide. There is growing evidence suggesting that excessive alcohol consumption can cause heart failure, liver cirrhosis and other organ damage, thereby indirectly causing kidney damage and impairing renal function (Rehm 2011; Varga et al. 2017; Yang et al. 2020b). Long-term alcohol consumption can directly lead to kidney damage through accelerated stone formation, endotoxin secretion, and fluid imbalance (Ojeda et al. 2012; Nasr et al. 2008; Bagshaw et al. 2005; Shankar et al. 2006).

Acute and chronic alcoholism can cause renal tubular hypofunction, as well as direct or indirect damage to the glomerulus and interstitium, eventually leading to renal failure. During alcoholic kidney damage, the prominent manifestation is renal tubular hypofunction. Ethanol mediates the effect of FTO on the PPAR-α m6A mRNA methylation in a YTHDF2-dependent manner. Importantly, alcohol also promotes the binding of YTHDF2 and PPAR-α and mediates their degradation. The reduction in PPAR-α ultimately leads to activation of the NLRP3 inflammasome and the release of downstream inflammatory cytokines, thereby enhancing renal inflammation and injury (Yu et al. 2021).

## Uremia

Patients with CKD have multiple cellular dysfunctions that lead to increased atherosclerosis, impaired immune function and metabolic disorders, which are in an end-stage called the uremic phase (Wong et al. 2012). It was found that indoxyl sulfate (uremic toxin) regulates leukocyte function by upregulating FTO levels. Decreasing m6A levels through the demethylation function of FTO affects leukocyte autophagy. Knockdown of FTO or inhibition of the m6A by 3-deazaadenosine blocks the effects of indoxyl sulfate on autophagy in cells. These findings provide new insights into the mechanisms of cellular dysfunction associated with CKD. Targeting RNA m6A modification may be a new strategy for the treatment of CKD (Wang et al. 2020).

## Obstructive nephropathy

Unilateral ureteral obstruction (UUO) is an important factor leading to renal impairment, which affects renal function mainly through damage to the renal tubules, glomeruli, etc. Even short-term UUO can lead to long-term renal insufficiency (Hammad 2022). Many studies have shown that m6A modifications are close related to the severity of renal interstitial fibrosis, and therefore some renal diseases can be treated by modulating RNA methylation (Feng et al. 2022). METTL3, METTL14 and FTO may play major roles in interstitial fibrosis during UUO nephropathy. Li et al. measured five enzymes associated with methylation modifications in UUO model mice and found that the downregulation of ALKBH5 and FTO and upregulation of METTL14 and METTL3 resulted in a time-specific increase in m6A levels in renal RNA. The authors also showed that epitranscriptional modifications in the TGF-β signaling pathway and axonal signaling pathway played an important role in obstructive interstitial fibrosis (Li et al. 2020). Li et al. found that FTO exacerbated renal interstitial fibrosis by reducing m6A modification of lncRNA GAS5. LncRNA GAS5 overexpression or FTO silencing suppressed the TGF-β1-induced increase in EMT-related protein (Vimentin, Snail and N-cadherin) and inflammatory cytokine (IL-6, IL-1β and TNF-α) levels in HK-2 cells. FTO can suppress lncRNA GAS5 expression via m6A modification to promote EMT and the inflammatory response (Li et al. 2022). Knockdown of ALKBH5 suppresses E-cadherin expression and promotes the levels of a-SMA and Snail, whereas overexpression of ALKBH5 exerted the opposite effects. Genistein could restore the expression of ALKBH5 to ameliorate renal fibrosis (Ning et al. 2020). Canagliflflozin (Cana), an SGLT2 inhibitor used to treat DN, is an important protein that protects the kidney from fibrosis by inhibiting FTO and increasing the stability of SQSTM1 (Yang et al. 2022).

METTL3 plays a crucial role in obstructive nephropathy, and one study revealed a future therapeutic strategy for obstructive nephropathy, in which METTL3 can promote MALAT1 expression, which was associated with binding and inhibiting miR-145, leading to the upregulation of FAK and ultimately to the exacerbation of renal fibrosis. This expression profile may be a biomarker for diagnosing renal fibrosis in obstructive nephropathy (Liu et al. 2020). Moreover, METTL3/*N*6-methyladenosine/miR-21-5p regulates inflammation through activation of the SPRY1/ERK/NF-κB pathway and promotes obstructive renal fibrosis. METTL3 and its reader protein HNRNPA2B1 promote miR-21-5p maturation. The increase in miR-21-5p promoted collagen I and FN synthesis and upregulated p-ERK1/2, p-NF-κB, IL-6 and TNF-α protein expression in HK-2 cells, while the expression of SPRY1 was inhibited (Liu et al. 2021b).

## Other kidney diseases

### Polycystic kidney disease

Autosomal dominant polycystic kidney disease (ADPKD) is one of the most common human monogenic diseases and is mainly caused by mutations in PKD1 or PKD2 (Patel et al. 2009; Ramalingma et al. 2020). ADPKD is typically characterized by a large number of tubular-derived cysts that increase in size over time, resulting in bilateral renal enlargement. Nearly 50% of patients progress to ESKD. Excessive cystic epithelial proliferation and abnormal cAMP and c-Myc signaling are key pathological hallmarks of ADPKD (Kurbegovic and Trudel 2020; Sussman et al. 2020; Wang et al. 2018). METTL3 can catalyze abundant m6A RNA modifications and participate in development. METTL3 deficiency or dietary methionine restriction was shown to improve ADPKD in a mouse model, providing some basis for treatment and remission. Elevated levels of methionine and *s*-adenosylmethionine (SAM) in the ADPKD model induced METTL3 expression and exacerbated isolated cyst growth, whereas dietary restriction of methionine attenuated ADPKD in mice. METTL3 promotes cyst proliferation and new protein synthesis by enhancing c-Myc and Avpr2 mRNA m6A modification and translational activation of the cyst-promoting c-Myc and cAMP pathway via c-Myc and cAMP signaling (Ramalingam et al. 2021).

### Podocytopathies

Podocytopathies are kidney diseases in which direct or indirect podocyte injury drives proteinuria or nephrotic syndrome (Kopp et al. 2020). Sirt1 is a protective deacetylase in proteinuric nephropathy, and studies show that METTL14 degrades Sirt1 mRNA by promoting its m6A modification, which decreases the stability of Sirt1 and thereby exacerbates podocyte injury and inflammatory responses (Lu et al. 2021). This provides an approach for the diagnosis and treatment of podocytopathies.

## Vascular calcification

Vascular calcification is closely associated with long-term alterations in mineral metabolism, inflammatory damage to vascular endothelial cells, secondary hyperparathyroidism and other related factors, such as methemoglobin A, in patients with CKD (Kaur and Singh 2022). Chen et al. found that vascular calcification was associated with METTL14. Klotho mRNA was demethylated when METTL14 expression was inhibited in human aortic smooth muscle cells, resulting in increased Klotho mRNA expression. The hypermethylation of Klotho mRNA and its association with decreased mRNA levels could be the result of METTL14 increasing the degradation of Klotho mRNA and other vascular-protecting mRNAs (Chen et al. 2019b). Increased METTL14 attenuates indoxyl sulfate-induced calcification, the loss of vasoprotective protein expression, and the loss of vascular repair function, thereby promoting vascular calcification and reducing vascular repair levels (Chen et al. 2019b).

## Summary and future directions

With in-depth studies of m6A methylation, researchers have increasingly focused on the role of m6A methylation in the clinical diagnosis and treatment of various diseases, which brings new insights into the pathogenesis of kidney disease. Studies have shown that methylation enzymes such as METTL14 and METTL3 may play a role in accelerating the progression of inflammation and promoting disease (Song et al. 2023), while demethylases such as FTO may slow disease progression through various pathways. Furthermore, several m6A methylation regulatory enzymes can be used as therapeutic targets or biomarkers of kidney diseases. For example, METTL3 can be used as a therapeutic target and as an early diagnostic indicator of kidney diseases such as ischemia‒reperfusion injury (Meng et al. 2020), cisplatin-induced AKI (Wang et al. 2022) and obstructive nephropathy (Liu et al. 2020); the demethylase FTO can be used as a therapeutic target for DN (Sun et al. 2022) and uremia (Wang et al. 2020). M6A methylation modification sites need to be recognized by reading proteins to function. For example, in renal injury due to podocyte damage, the authors identified a role for m6A binding proteins, particularly YTHDF2, in Sirt1 downregulation mediated by METTL14 under pathological conditions. To date, few studies of noncancer pathogenesis have addressed the important role of reading proteins. Therefore, exploring how m6A reading proteins play a role in kidney diseases may be a worthwhile direction for future research.

There are currently 24 known m6A regulatory proteins, but studies on m6A methylation in kidney disease have been limited to four to five enzymes such as METTL14, METTL3 and FTO, and there have been few studies on other enzymes. Moreover, METTL3 is more closely associated with kidney disease than METTL14 and FTO, although there are already related drugs to improve renal function and treat renal diseases through METTL3, more in-depth research and exploration can be carried out in this aspect in the future.

In summary, m6A modification plays an important role in acute and chronic kidney diseases. We look forward to further studies to elucidate the role and detailed mechanism of m6A methylation in the pathogenesis of kidney disease. This review aimed to provide new ideas for the diagnosis, treatment and prognosis of kidney disease.

## Data Availability

Not applicable.

## Abbreviations

m6A: *N*6-Methyladenosine
UTRs: 3ʹ-Untranslated regions
YTHDF: YTH N6-methyladenosine RNA binding proteins
PolyA: Polyadenylate
mRNA: Messenger RNA
miRNA: Micro RNA
circRNA: Circular RNA
lncRNA: Long noncoding RNA
METTL3: Methyltransferase Like 3
METTL14: Methyltransferase Like 14
WTAP: Recombinant Wilms Tumor 1 Associated Protein
FTO: Fat mass and obesity-associated protein
AKI: Acute kidney disease
EMT: Epithelial–mesenchymal transition
IRI: Ischemia reperfusion injury
DN: Diabetic nephropathy
LN: Lupus nephritis
SLE: Systemic lupus erythematosus
UUO: Unilateral ureteral obstructive nephropathy
ADPKD: Autosomal dominant polycystic kidney disease
ESKD: End-stage kidney disease

## References

[CR1] Anders HJ, Saxena R, Zhao MH, Parodis I, Salmon JE, Mohan C (2020). Lupus nephritis. Nat Rev Dis Primers.

[CR2] Bagshaw SM, Laupland KB, Doig CJ, Mortis G, Fick GH, Mucenski M (2005). Prognosis for long-term survival and renal recovery in critically ill patients with severe acute renal failure: a population-based study. Crit Care.

[CR3] Bonventre JV, Yang L (2011). Cellular pathophysiology of ischemic acute kidney injury. J Clin Invest.

[CR4] Bonventre JV, Vaidya VS, Schmouder R, Feig P, Dieterle F (2010). Next-generation biomarkers for detecting kidney toxicity. Nat Biotechnol.

[CR5] Chen J, Ning Y, Zhang H, Song N, Gu Y, Shi Y (2019). METTL14-dependent m6A regulates vascular calcification induced by indoxyl sulfate. Life Sci.

[CR6] Chen XY, Zhang J, Zhu JS (2019). The role of m(6)A RNA methylation in human cancer. Mol Cancer.

[CR7] Chen J, Xu C, Yang K, Gao R, Cao Y, Liang L (2023). Inhibition of ALKBH5 attenuates I/R-induced renal injury in male mice by promoting Ccl28 m6A modification and increasing Treg recruitment. Nat Commun.

[CR8] Choudhury D, Tuncel M, Levi M (2010). Diabetic nephropathy—a multifaceted target of new therapies. Discov Med.

[CR9] Cui YH, Yang S, Wei J, Shea CR, Zhong W, Wang F (2021). Autophagy of the m6A mRNA demethylase FTO is impaired by low-level arsenic exposure to promote tumorigenesis. Nat Commun.

[CR10] Desrosiers R, Friderici K, Rottman F (1974). Identification of methylated nucleosides in messenger RNA from Novikoff hepatoma cells. Proc Natl Acad Sci U S A.

[CR11] Dominissini D, Moshitch-Moshkovitz S, Schwartz S, Salmon-Divon M, Ungar L, Osenberg S (2012). Topology of the human and mouse m6A RNA methylomes revealed by m6A-seq. Nature.

[CR12] Feng C, Wang Z, Liu C, Liu S, Wang Y, Zeng Y (2022). Integrated bioinformatical analysis, machine learning and *in vitro* experiment-identified m6A subtype, and predictive drug target signatures for diagnosing renal fibrosis. Front Pharmacol.

[CR13] Gu HF (2019). Genetic and epigenetic studies in diabetic kidney disease. Front Genet.

[CR14] Hammad FT (2022). The long-term renal effects of short periods of unilateral ureteral obstruction. Int J Physiol Pathophysiol Pharmacol.

[CR15] Han D, Liu J, Chen C, Dong L, Liu Y, Chang R (2019). Anti-tumour immunity controlled through mRNA m6A methylation and YTHDF1 in dendritic cells. Nature.

[CR16] Hu J, Lieb JD, Sancar A, Adar S (2016). Cisplatin DNA damage and repair maps of the human genome at single-nucleotide resolution. Proc Natl Acad Sci U S A.

[CR17] Hu J, Qiu D, Yu A, Hu J, Deng H, Li H (2021). YTHDF1 is a potential pan-cancer biomarker for prognosis and immunotherapy. Front Oncol.

[CR18] Hu J, Wang Q, Fan X, Zhen J, Wang C, Chen H (2023). Long noncoding RNA ENST00000436340 promotes podocyte injury in diabetic kidney disease by facilitating the association of PTBP1 with RAB3B. Cell Death Dis.

[CR19] Jiang L, Li X, Wang S, Yuan Z, Cheng J (2022). The role and regulatory mechanism of m6A methylation in the nervous system. Front Genet.

[CR20] Jiang L, Liu X, Hu X, Gao L, Zeng H, Wang X (2022). METTL3-mediated m6A modification of TIMP2 mRNA promotes podocyte injury in diabetic nephropathy. Mol Ther.

[CR21] Kaur R, Singh R (2022). Mechanistic insights into CKD-MBD-related vascular calcification and its clinical implications. Life Sci.

[CR22] Kopp JB, Anders HJ, Susztak K, Podestà MA, Remuzzi G, Hildebrandt F (2020). Podocytopathies. Nat Rev Dis Primers.

[CR23] Kumari R, Ranjan P, Suleiman ZG, Goswami SK, Li J, Prasad R (2022). mRNA modifications in cardiovascular biology and disease: with a focus on m6A modification. Cardiovasc Res.

[CR24] Kurbegovic A, Trudel M (2020). The master regulators Myc and p53 cellular signaling and functions in polycystic kidney disease. Cell Signal.

[CR25] Li X, Fan X, Yin X, Liu H, Yang Y (2020). Alteration of N6-methyladenosine epitranscriptome profile in unilateral ureteral obstructive nephropathy. Epigenomics.

[CR26] Li C, Jiang Z, Hao J, Liu D, Hu H, Gao Y (2021). Role of N6-methyl-adenosine modification in mammalian embryonic development. Genet Mol Biol.

[CR27] Li CM, Li M, Zhao WB, Ye ZC, Peng H (2021). Alteration of N6-methyladenosine RNA profiles in cisplatin-induced acute kidney injury in mice. Front Mol Biosci.

[CR28] Li M, Deng L, Xu G (2021). METTL14 promotes glomerular endothelial cell injury and diabetic nephropathy via m6A modification of α-klotho. Mol Med.

[CR29] Li N, Tang H, Wu L, Ge H, Wang Y, Yu H (2021). Chemical constituents, clinical efficacy and molecular mechanisms of the ethanol extract of Abelmoschus manihot flowers in treatment of kidney diseases. Phytother Res.

[CR30] Li X, Jiang Y, Sun X, Wu Y, Chen Z (2021). METTL3 is required for maintaining β-cell function. Metabolism.

[CR31] Li X, Li Y, Wang Y, He X (2022). The m6A demethylase FTO promotes renal epithelial-mesenchymal transition by reducing the m6A modification of lncRNA GAS5. Cytokine.

[CR32] Liu J, Yue Y, Han D, Wang X, Fu Y, Zhang L (2014). A METTL3-METTL14 complex mediates mammalian nuclear RNA N6-adenosine methylation. Nat Chem Biol.

[CR33] Liu P, Zhang B, Chen Z, He Y, Du Y, Liu Y (2020). m6A-induced lncRNA MALAT1 aggravates renal fibrogenesis in obstructive nephropathy through the miR-145/FAK pathway. Aging (albany NY).

[CR34] Liu BH, Tu Y, Ni GX, Yan J, Yue L, Li ZL (2021). Total flavones of *Abelmoschus manihot* ameliorates podocyte pyroptosis and injury in high glucose conditions by targeting METTL3-dependent m6A modification-mediated NLRP3-inflammasome activation and PTEN/PI3K/Akt signaling. Front Pharmacol.

[CR35] Liu E, Lv L, Zhan Y, Ma Y, Feng J, He Y (2021). METTL3/N6-methyladenosine/ miR-21-5p promotes obstructive renal fibrosis by regulating inflammation through SPRY1/ERK/NF-κB pathway activation. J Cell Mol Med.

[CR36] Liu B, Ao S, Tan F, Ma W, Liu H, Liang H (2022). Transcriptomic analysis and laboratory experiments reveal potential critical genes and regulatory mechanisms in sepsis-associated acute kidney injury. Ann Transl Med.

[CR37] Liu T, Zhuang XX, Qin XJ, Wei LB, Gao JR (2023). The potential role of N6-methyladenosine modification of LncRNAs in contributing to the pathogenesis of chronic glomerulonephritis. Inflamm Res.

[CR38] Livneh I, Moshitch-Moshkovitz S, Amariglio N, Rechavi G, Dominissini D (2020). The m6A epitranscriptome: transcriptome plasticity in brain development and function. Nat Rev Neurosci.

[CR39] Loghman-Adham M, Kiu Weber CI, Ciorciaro C, Mann J, Meier M (2012). Detection and management of nephrotoxicity during drug development. Expert Opin Drug Saf.

[CR40] Lu Z, Liu H, Song N, Liang Y, Zhu J, Chen J (2021). METTL14 aggravates podocyte injury and glomerulopathy progression through N6-methyladenosine-dependent downregulating of Sirt1. Cell Death Dis.

[CR41] Luo S, Tong L (2014). Molecular basis for the recognition of methylated adenines in RNA by the eukaryotic YTH domain. Proc Natl Acad Sci U S A.

[CR42] Majumder S, Hadden MJ, Thieme K, Batchu SN, Niveditha D, Chowdhury S (2019). Dysregulated expression but redundant function of the long non-coding RNA HOTAIR in diabetic kidney disease. Diabetologia.

[CR43] Mao Y, Jiang F, Xu XJ, Zhou LB, Jin R, Zhuang LL (2023). Inhibition of IGF2BP1 attenuates renal injury and inflammation by alleviating m6A modifications and E2F1/MIF pathway. Int J Biol Sci.

[CR44] Meng F, Liu Y, Chen Q, Ma Q, Gu S, Cui R (2020). METTL3 contributes to renal ischemia-reperfusion injury by regulating Foxd1 methylation. Am J Physiol Renal Physiol.

[CR45] Nasr SH, Markowitz GS, Stokes MB, Said SM, Valeri AM, D'Agati VD (2008). Acute postinfectious glomerulonephritis in the modern era: experience with 86 adults and review of the literature. Medicine (baltimore).

[CR46] Ning Y, Chen J, Shi Y, Song N, Yu X, Fang Y (2020). Genistein ameliorates renal fibrosis through regulation snail via m6A RNA demethylase ALKBH5. Front Pharmacol.

[CR47] Ojeda ML, Barrero MJ, Nogales F, Murillo ML, Carreras O (2012). Oxidative effects of chronic ethanol consumption on the functions of heart and kidney: folic acid supplementation. Alcohol Alcohol.

[CR48] Patel V, Chowdhury R, Igarashi P (2009). Advances in the pathogenesis and treatment of polycystic kidney disease. Curr Opin Nephrol Hypertens.

[CR49] Peerapornratana S, Manrique-Caballero CL, Gómez H, Kellum JA (2019). Acute kidney injury from sepsis: current concepts, epidemiology, pathophysiology, prevention and treatment. Kidney Int.

[CR50] Ping XL, Sun BF, Wang L, Xiao W, Yang X, Wang WJ (2014). Mammalian WTAP is a regulatory subunit of the RNA N6-methyladenosine methyltransferase. Cell Res.

[CR51] Ramalingam H, Yheskel M, Patel V (2020). Modulation of polycystic kidney disease by non-coding RNAs. Cell Signal.

[CR52] Ramalingam H, Kashyap S, Cobo-Stark P, Flaten A, Chang CM, Hajarnis S (2021). A methionine-Mettl3-N6-methyladenosine axis promotes polycystic kidney disease. Cell Metab.

[CR53] Rehm J (2011). The risks associated with alcohol use and alcoholism. Alcohol Res Health J Natl Inst Alcohol Abuse Alcohol.

[CR54] Rottman F, Shatkin AJ, Perry RP (1974). Sequences containing methylated nucleotides at the 5' termini of messenger RNAs: possible implications for processing. Cell.

[CR55] Schafer KP (1982). RNA synthesis and processing reactions in a subcellular system from mouse L cells. Hoppe Seylers Z Physiol Chem.

[CR56] Shankar A, Klein R, Klein BE (2006). The association among smoking, heavy drinking, and chronic kidney disease. Am J Epidemiol.

[CR57] Shen J, Wang W, Shao X, Wu J, Li S, Che X (2020). Integrated analysis of m6A methylome in cisplatin-induced acute kidney injury and berberine alleviation in mouse. Front Genet.

[CR58] Song B, Zeng Y, Cao Y, Zhang J, Xu C, Pan Y (2023). Emerging role of METTL3 in inflammatory diseases: mechanisms and therapeutic applications. Front Immunol.

[CR59] Sun Q, Geng H, Zhao M, Li Y, Chen X, Sha Q (2022). FTO-mediated m6 A modification of SOCS1 mRNA promotes the progression of diabetic kidney disease. Clin Transl Med.

[CR60] Sussman CR, Wang X, Chebib FT, Torres VE (2020). Modulation of polycystic kidney disease by G-protein coupled receptors and cyclic AMP signaling. Cell Signal.

[CR61] Tang W, Zhao Y, Zhang H, Peng Y, Rui Z (2022). METTL3 enhances NSD2 mRNA stability to reduce renal impairment and interstitial fibrosis in mice with diabetic nephropathy. BMC Nephrol.

[CR62] Varga ZV, Matyas C, Paloczi J, Pacher P (2017). Alcohol misuse and kidney injury: epidemiological evidence and potential mechanisms. Alcohol Res.

[CR63] Wang Q, Cobo-Stark P, Patel V, Somlo S, Han PL, Igarashi P (2018). Adenylyl cyclase 5 deficiency reduces renal cyclic AMP and cyst growth in an orthologous mouse model of polycystic kidney disease. Kidney Int.

[CR64] Wang J, Ishfaq M, Xu L, Xia C, Chen C, Li J (2019). METTL3/m6A/miRNA-873-5p attenuated oxidative stress and apoptosis in colistin-induced kidney injury by modulating Keap1/Nrf2 pathway. Front Pharmacol.

[CR65] Wang CY, Lin TA, Ho MY, Yeh JK, Tsai ML, Hung KC (2020). Regulation of autophagy in leukocytes through RNA N6-adenosine methylation in chronic kidney disease patients. Biochem Biophys Res Commun.

[CR66] Wang JN, Wang F, Ke J, Li Z, Xu CH, Yang Q (2022). Inhibition of *METTL3* attenuates renal injury and inflammation by alleviating *TAB3* m6A modifications via IGF2BP2-dependent mechanisms. Sci Transl Med.

[CR67] Wertheim H, Van Nguyen K, Hara GL, Gelband H, Laxminarayan R, Mouton J (2013). Global survey of polymyxin use: a call for international guidelines. J Glob Antimicrob Resist.

[CR68] Wong CJ, Moxey-Mims M, Jerry-Fluker J, Warady BA, Furth SL (2012). CKiD (CKD in children) prospective cohort study: a review of current findings. Am J Kidney Dis.

[CR69] Xu Y, Yuan XD, Wu JJ, Chen RY, Xia L, Zhang M (2020). The N6-methyladenosine mRNA methylase METTL14 promotes renal ischemic reperfusion injury via suppressing YAP1. J Cell Biochem.

[CR70] Xu Z, Jia K, Wang H, Gao F, Zhao S, Li F (2021). METTL14-regulated PI3K/Akt signaling pathway via PTEN affects HDAC5-mediated epithelial-mesenchymal transition of renal tubular cells in diabetic kidney disease. Cell Death Dis.

[CR71] Yang Y, Fan X, Mao M, Song X, Wu P, Zhang Y (2017). Extensive translation of circular RNAs driven by N6-methyladenosine. Cell Res.

[CR72] Yang C, Wang H, Zhao X, Matsushita K, Coresh J, Zhang L (2020). CKD in China: evolving spectrum and public health implications. Am J Kidney Dis.

[CR73] Yang Q, Chen HY, Wang JN, Han HQ, Jiang L, Wu WF (2020). Alcohol promotes renal fibrosis by activating Nox2/4-mediated DNA methylation of Smad7. Clin Sci (lond).

[CR74] Yang Y, Li Q, Ling Y, Leng L, Ma Y, Xue L (2022). m6A eraser FTO modulates autophagy by targeting SQSTM1/P62 in the prevention of canagliflozin against renal fibrosis. Front Immunol.

[CR75] Yu JT, Hu XW, Chen HY, Yang Q, Li HD, Dong YH (2021). DNA methylation of FTO promotes renal inflammation by enhancing m6A of PPAR-α in alcohol-induced kidney injury. Pharmacol Res.

[CR76] Zhao H, Pan S, Duan J, Liu F, Li G, Liu D (2021). Integrative analysis of m6A regulator-mediated RNA methylation modification patterns and immune characteristics in lupus nephritis. Front Cell Dev Biol.

[CR77] Zheng G, Dahl JA, Niu Y, Fedorcsak P, Huang CM, Li CJ (2013). ALKBH5 is a mammalian RNA demethylase that impacts RNA metabolism and mouse fertility. Mol Cell.

[CR78] Zhou P, Wu M, Ye C, Xu Q, Wang L (2019). Meclofenamic acid promotes cisplatin-induced acute kidney injury by inhibiting fat mass and obesity-associated protein-mediated m6A abrogation in RNA. J Biol Chem.

[CR79] Zhu S, Lu Y (2020). Dexmedetomidine suppressed the biological behavior of HK-2 cells treated with LPS by down-regulating ALKBH5. Inflammation.

